# Effects of Capsinoid Intake on Brown Adipose Tissue Vascular Density and Resting Energy Expenditure in Healthy, Middle-Aged Adults: A Randomized, Double-Blind, Placebo-Controlled Study

**DOI:** 10.3390/nu12092676

**Published:** 2020-09-02

**Authors:** Sayuri Fuse, Tasuki Endo, Riki Tanaka, Miyuki Kuroiwa, Akira Ando, Ayami Kume, Akiko Yamamoto, Kanna Kuribayashi, Shinji Somekawa, Masamichi Takeshita, Masaki Hashimoto, Ryotaro Kime, Yuko Kurosawa, Takafumi Hamaoka

**Affiliations:** 1 Department of Sports Medicine for Health Promotion, Tokyo Medical University, 6-1-1 Shinjuku, Shinjuku-ku, Tokyo 160-8402, Japan; fsayuri.65@gmail.com (S.F.); endo@tokyo-med.ac.jp (T.E.); s118042@tokyo-med.ac.jp (R.T.); mkuroiwa@tokyo-med.ac.jp (M.K.); axsry@yahoo.co.jp (A.K.); kime@tokyo-med.ac.jp (R.K.); yuko.kurosawa.2011@gmail.com (Y.K.); 2 Department of Sport Sciences, Japan Institute of Sports Sciences, 3-15-1 Nishigaoka, Kita-ku, Tokyo 115-0056, Japan; aand2.x2@gmail.com; 3 Ajinomoto Co., Inc., Institute of Food Sciences and Technologies, 1-1 Suzuki-cho, Kawasaki-ku, Kawasaki City, Kanagawa 210-8681, Japan; akiko_yamamoto01@ajinomoto.com (A.Y.); kanna_kuribayashi@ajinomoto.com (K.K.); shinji_somekawa@ajinomoto.com (S.S.); 4 Ajinomoto Co., Inc., Research Institute for Bioscience Products & Fine Chemicals, 1-1 Suzuki-cho, Kawasaki-ku, Kawasaki City, Kanagawa 210-8681, Japan; masamichi_takeshita@ajinomoto.com; 5 Ajinomoto Co., Inc., Direct Marketing Department, 15-1, Kyobashi 1-chome, Chuo-ku, Tokyo 104-8315, Japan; masaki_hashimoto@ajinomoto.com

**Keywords:** capsinoids, dihydrocapsiate, energy expenditure, brown adipose tissue, middle-aged, near-infrared time-resolved spectroscopy

## Abstract

Capsinoids are some of the most promising ingredients to increase energy expenditure (EE) due to brown adipose tissue (BAT) activation. However, there is limited information regarding the effect of prolonged capsinoid ingestion (CI) on BAT activity and resting EE (REE) in healthy, middle-aged, normal to overweight subjects (Sub_healthy_) with distinct BAT characteristics. We examined the changes in BAT density (BAT-d), using near-infrared time-resolved spectroscopy, and REE/kg induced by daily CI. Forty Sub_healthy_ [age, 43.8 (mean) years; BMI, 25.4 kg/m^2^] received either capsinoid (9 mg/day) or a placebo daily for 6 weeks in a double-blind design. Total hemoglobin concentration in the supraclavicular region ([total-Hb]_sup_), an indicator of BAT-d, and REE/kg were measured. The changes in post-intervention [total-Hb]_sup_ were greater in the capsinoid group (CA-G) than in the placebo group (PL-G) [5.8 µM (+12.4%) versus 1.0 µM (+2.1%); *p* = 0.017]. There was a significant relationship between BAT-d and REE/kg; however, post-supplementation REE/kg was not significantly different between the two groups (*p* = 0.228). In the overweight subgroup, changes in REE/kg were greater in the CA-G than in the PL-G [0.6 cal/kg/min (+4.3%) versus −0.3 cal/kg/min (−2.1%); *p* = 0.021]. CI enhanced [total-Hb]_sup_, a reflection of BAT-d, showing a good correlation with REE in Sub_healthy_.

## 1. Introduction

Brown adipose tissue (BAT) is known to promote nonshivering thermogenesis during cold exposure and spontaneous hyperphagia in animal models [1]. Recently, noninvasive, non-ionizing-radiation near-infrared time-resolved spectroscopy (NIR_TRS_) has been reported as an alternative method for measuring BAT vascular density (BAT-d), which is equivalent to the BAT activity or amount of BAT in humans [2,3,4]. Human BAT is reported to be related to body fat [5,6,7], participate in glucose homeostasis [8,9], and improve blood lipid profile [10,11]. Thus, BAT is a potentially preventive and therapeutic agent in combating obesity and lifestyle-related diseases.

Certain food ingredients such as capsaicin and capsinoids stimulate BAT by the same mechanism as that of cold exposure [12]. It has been reported that capsinoids activate transient potential receptor (TRP) vanilloid 1 (TRPV1), increase sympathetic nervous system (SNS) activity and energy expenditure (EE), and potentiate the decrease in body fat in humans [13,14]. Further reports have noted that the binding affinity for TRPV1, increase of plasma catecholamine concentration, and enhancement of oxygen consumption (VO_2_) were comparable among three components of capsinoids [15], namely, capsiate, dihydrocapsiate, and nordihydrocapsiate, found in a nonpungent type of red pepper called “CH-19 Sweet” (*Capsicum annuum* L.) [11,15,16]. Capsiate obtains an unsaturated hydrocarbon bond and consequently becomes chemically unstable; therefore, dihydrocapsiate and nordihydrocapsiate would be preferable candidates owing to their chemical stability. Further, dihydrocapsiate was used in this study because the amount of dihydrocapsiate present in CH-19 Sweet is greater than that of nordihydrocapsiate.

In previous studies conducted on young, lean individuals, the acute effect of capsinoid ingestion has significantly increased resting EE (REE) only in metabolically active BAT [17,18,19]. However, there was no significant increase in REE by acute capsinoid intake in middle-aged obese populations [13] or young, overweight individuals [20] whose BAT activity was unclear. In addition, prolonged capsinoid ingestion by individuals with unknown BAT activity has resulted in significant reduction in body weight and suppression of body fat accumulation in young, lean populations [21] as well as increase in REE in overweight to obese, middle-aged individuals [16,22]. Collectively, there is limited information available regarding the effect of prolonged capsinoid ingestion on BAT-d and REE, and their relationships in the middle-aged to elderly, normal to overweight population with distinct BAT characteristics.

Therefore, this study aimed to confirm whether prolonged ingestion of thermogenic capsinoid increases BAT-d and REE in normal to overweight, middle-aged adults.

## 2. Materials and Methods

### 2.1. Study Design

This randomized, double-blind, placebo-controlled study was conducted between 17 November 2019 and 16 February 2020, (late fall to winter) in Japan. The participants received either dihydrocapsiate [9 mg/day; capsinoid group (CA-G)] or placebo [placebo group (PL-G)] capsules daily for 6 weeks. The intervention started between 15 December 2019 and 5 January 2020 and ended between 26 January and 16 February 2020 for the CA-G. For the PL-G, the intervention started between 15 December 2019 and 4 January 2020 and ended between 26 January and 15 February 2020. Their anthropometric and circulatory parameters, BAT-d, and resting VO_2_ in muscle and subcutaneous fat were measured every 3 weeks. In addition, before and after the 6-week intervention, their REE and SNS activity were measured. The participants were instructed to maintain their usual dietary intake and physical activity during the experimental period. The study design and protocol were approved by the Institutional Review Board of Tokyo Medical University (T2019-0002) and Ajinomoto Co., Inc., in accordance with the ethical principles defined in the Declaration of Helsinki. Written informed consent was obtained from all participants. This trial was registered with the University Hospital Medical Information Network (UMIN000038121).

### 2.2. Participants

The participants were recruited through posting advertisements on posters, Internet, or direct contact. Enrollment targeted 40 healthy male participants [age, 30–64 years; body mass index (BMI), 23–28 kg/m^2^], who could ingest a capsule as well as present themselves at Tokyo Medical University where the examination was conducted. Females were not eligible for this study because we cannot accurately evaluate their EE because of menstrual cycles. Primary exclusion criteria were as follows: the routine intake of nutritionally functional food or supplements and/or medicine that has effects on energy metabolism or thermogenesis (e.g., catechin, sesamin, capsaicin, monoglucosyl hesperidin, coenzyme Q10, and adenosine triphosphate), extremely high BAT-d of greater than 100 µM of total hemoglobin concentration at the supraclavicular region ([total-Hb]_sup_), obesity, and hyperthyroidism-related diseases. According to our previous study on the relationship between [total-Hb]_sup_ and positron emission tomography combined with computed tomography (PET/CT)-determined ^18^F-fluorodeoxyglucose (^18^FDG) uptake in the supraclavicular region, a [total-Hb]_sup_ value over 74 µM corresponded to BAT positive values (^18^FDG uptake over 2.0) [2]. We suspected that subjects with extremely high [total-Hb]_sup_ (74 µM + 2 SD ≈ 100 µM) might not experience an increase in [total-Hb]_sup_ through capsinoid supplementation because these subjects had a genetic predisposition or were exposed to certain environmental stimuli to potentiate BAT activation. The participants were randomly allocated to the CA-G or PL-G by a non-participating third party.

### 2.3. Capsinoid Supplementation

Dihydrocapsiate, a capsinoid compound, can be extracted from CH-19 Sweet. In this study, dihydrocapsiate was enzymatically synthesized using vanillyl alcohol and 8-methylnonanoic acid through esterification, filtration, extraction, and evaporation. Refined rapeseed oil was used to dilute dihydrocapsiate to a concentration of 2.48%. Capsules of 4.5 mg dihydrocapsiate were then produced. The placebo capsules, containing refined rapeseed oil, were prepared in the same manner. Both capsules were supplied by Ajinomoto Co., Inc. (Tokyo, Japan). Each participant was instructed to take one capsule between the hour of rising and before breakfast and between after dinner and retiring for the night (a total of 9 mg/day orally); they were instructed to register daily intake through a diary for 6 weeks. The average supplemental compliance rate (completed intake times/designated intake times per a person) was 99.3%.

### 2.4. Outcomes

The primary endpoints were the BAT-d and REE values after the 6-week capsinoid treatment, and the secondary endpoints were changes in anthropometric and circulatory parameters, resting VO_2_ in muscle and subcutaneous fat, and SNS activity pre- and post-treatment. An exploratory subgroup analysis was conducted on the REE values in participants whose BMI was greater than 25 kg/m^2^.

### 2.5. Anthropometric and Circulatory Measurements

Body weight was measured using bioelectric impedance (Inbody 720 Body Composition Analyzer; InBody Japan, Tokyo, Japan). BMI was calculated as follows: body weight in kilograms divided by the square of height in meters (kg/m^2^). Visceral adipose tissue area (VATA) was estimated using bioelectrical impedance analysis (EW-FA90; Panasonic, Osaka, Japan). Systolic and diastolic blood pressures as well as heart rate were measured using an automated sphygmomanometer (HEM-1025; Omron Healthcare, Kyoto, Japan).

### 2.6. BAT-d Measurements

We confirmed that [total-Hb]_sup_ was not different between temperatures of 19 °C and 27 °C [2]. The [total-Hb]_sup_, a parameter of BAT-d, was measured using NIR_TRS_ (TRS-20; Hamamatsu Photonics K.K., Hamamatsu, Japan) for 1 min at 23–25 °C in the same way as mentioned in a previous study measuring REE [23]. The probes were placed on the skin of the supraclavicular region, an area that contains BAT. Participants were required to remain in the sitting position, wearing light clothing, such as T-shirts, during measurements, as previously described [2,7,24,25,26,27]. Compared to visible light wavelengths, NIR wavelengths (700–3000 nm) show less scattering and, consequently, better penetration into the biological tissue. However, light absorption by water limits tissue penetration at wavelengths above 900 nm; thus, a 650–900 nm range is suitable for measurements [28]. Accordingly, we used NIR wavelengths of 760, 800, and 830 nm to evaluate oxygenated hemoglobin (oxy-Hb), deoxygenated hemoglobin (deoxy-Hb), and total-Hb concentrations, respectively. With the 3-cm probe used in this study, light can reach a mean depth of 2 cm [29], where BAT is potentially located [30]. Among these NIR_TRS_ parameters, [total-Hb]_sup_ has been examined for assessing BAT-d as a potential parameter of blood volume (or tissue vasculature density) [2]. Specifically, vascular density is higher in BAT than in white adipose tissue (WAT) [31]. The [total-Hb]_sup_ measures under both thermoneutral and cold conditions were positively correlated with parameters determined by ^18^FDG-PET/CT with cold exposure in the supraclavicular region, but not in the deltoid muscle region (control site) [2]. Further, a significant correlation was reported between cold-induced thermogenesis and [total-Hb]_sup_ in winter [3]. Thus, [total-Hb]_sup_ determined by NIR_TRS_ could be a reasonable alternative to BAT activity determined by ^18^FDG-PET/CT, which has several limitations, including enormous instrumentation costs, ionizing radiation exposure, and acute cold exposure [32].

The tissue was illuminated using a 200-µm core diameter optical fiber by the light generated from 100 ps full-width at half-maximum optic pulses, at a 5-MHz repetition rate and an average power of 80 µW for each wavelength. The emitted photons penetrated the tissue and were reflected to a 3-mm diameter optical bundle fiber, through which they were sent to a photomultiplier tube for single-photon detection and a signal-processing circuit for time-resolved measurement. Using the nonlinear least-squares method, the digitized temporal profile data from in vitro samples or tissue were fitted with a theoretical temporal profile, derived from the analytical solution of the photon diffusion theory with a semi-infinite homogeneous reflectance model. After convolution with the instrumental response function, to compensate for the time response of the instrument itself, absorption coefficient and reduced scattering coefficient values at 760, 800, and 830 nm were obtained using the least-squares fitting method. Thereafter, the absolute total-Hb concentration was calculated as the sum of oxy-Hb and deoxy-Hb concentrations [28]. The NIR_TRS_ system collected data every 10 s. The coefficient of variation for repeated measurements of the total-Hb concentration was 4.9% [2].

### 2.7. Resting Energy Expenditure

REE was estimated using a respiratory gas analyzer (AE310S, Minato Medical Science, Osaka, Japan) in the morning. Subjects were instructed to undergo an overnight fast of 10–13 h and not to perform any vigorous exercise within 24 h before measurement. Upon arrival at the Tokyo Medical University laboratory, subjects rested quietly in bed in the supine position for 20 min under room temperature (23–25 °C). Thereafter, pulmonary VO_2_ and carbon dioxide production (VCO_2_) were continually recorded for 10 min. The stable value during the 8-min period between the first and the last minute was used to calculate REE per kg (REE/kg). REE/kg was calculated using the following formula:

REE/kg (cal/kg/min) = 3.9 × VO_2_ (ml/kg/min) + 1.1 × VCO_2_ (ml/kg/min) (1)

### 2.8. Resting Oxygen Consumption Rate in the Muscle and Subcutaneous Fat

Normally, we do need a six-minute arterial occlusion method to calibrate zero oxygenation when using continuous wave NIRS, which does not provide absolute values [33]. In this study, as NIR_TRS_ was used, which provides absolute values (µM O_2_/s), the zeroing calibration was not required [34]. Resting VO_2_ rate in the muscle and subcutaneous fat was evaluated by the brief arterial occlusion method using NIR_TRS_ [28,35]. Arterial occlusion of the right upper arm was performed by inflating the cuff tourniquet to a pressure of 300 mmHg for 3 min. Subjects held the right elbow at 90° of flexion, in line with the level of the heart, while seated during measurement. The probe with a light source-detector separation of 3 cm was placed on the skin above the musculus flexor carpi ulnaris to evaluate muscle VO_2_ and that of 1 cm was placed on the skin with underlying subcutaneous fat, of which the skin surrounding the elbow had the thickest. The skin with underlying subcutaneous fat was pinched and heaped up using soft clothespins for the accurate measurement of fat VO_2_. The initial decline rate of oxy-Hb concentration minus deoxy-Hb for 2 min following the onset of occlusion was calculated using the linear regression as an index of resting VO_2_.

### 2.9. Sympathetic Activity

Pulse rate variability (PRV) measurements are used to noninvasively estimate autonomic nervous system (ANS) function [36,37], together with those of heart rate variability [38]. The PRV frequency domain method can distinguish high-frequency (HF > 25 Hz) components that purely reflect parasympathetic nervous system activity from low-frequency (LF < 0.15 Hz) and very low-frequency (0.003–0.15 Hz) components that reflect both sympathetic [39] and parasympathetic nervous system activities [40].

Subjects who measured their PRV frequency by placing a sphygmograph (TAS9 Pulse Analyzer Plus; YKC, Tokyo, Japan) on their left index fingertip did so in the supine position for 15 min. The PRV frequency domain data were automatically analyzed using a fast Fourier transform [41,42]. This analysis involved 1000 samples, a 300-ms pulse-interval re-sampling frequency, and Hanning window function. The correspondence of PRV with heart rate variability in the resting state is well documented [36,43]. The LF and HF components were defined as the areas under the spectral peaks within the ranges of 0.04–0.15 Hz and 0.15–0.4 Hz, respectively. We assessed SNS activity by calculating the LF-to-HF ratio (LF/HF) [25].

### 2.10. Statistical Analysis

Sample size was calculated considering the type 1 and type 2 errors based on the statistical testing of BAT-d, which was one of the primary outcomes. With reference to a previous double-blinded parallel-group study that examined changes of BAT-d in healthy adults who ingested capsinoids or placebo capsules for 8 weeks [27], the amount of change in BAT-d was estimated as 31.8 ± 21.7 [mean ± standard deviation (SD)] µM and 10.6 ± 21.7 µM for the CA-G and PL-G, respectively. In this setting, sample size was calculated at 80% power and 5% significance level. This resulted in a net sample size of 18 subjects in each group. Therefore, we decided to enroll 20 subjects in each group in case of two dropouts.

Data were expressed as mean ± SD. The BAT-d and REE/kg values were analyzed using repeated measures analysis of variance (ANOVA) including “time” (weeks 0, 3, and 6) which was considered a “within subject” factor, and PL-G and CA-G which were considered “between subject” factors. If a significant interaction or main effect was observed, a post hoc comparison was conducted by using Bonferroni’s test. The Welch test was conducted to assess the amount of changes from baseline. Moreover, we conducted similar additional analyses of REE/kg values for the subgroups with BMI over 25 kg/m^2^. For the other outcome measures, the Welch test was used again to test the significant difference between groups. Pearson’s correlation analysis was used to evaluate the relationship between BAT-d and REE/kg. Values were considered to indicate statistical significance if *p* < 0.05. All statistical analyses were performed using SPSS version 26 (IBM Japan, Tokyo, Japan).

## 3. Results

**Figure 1:**
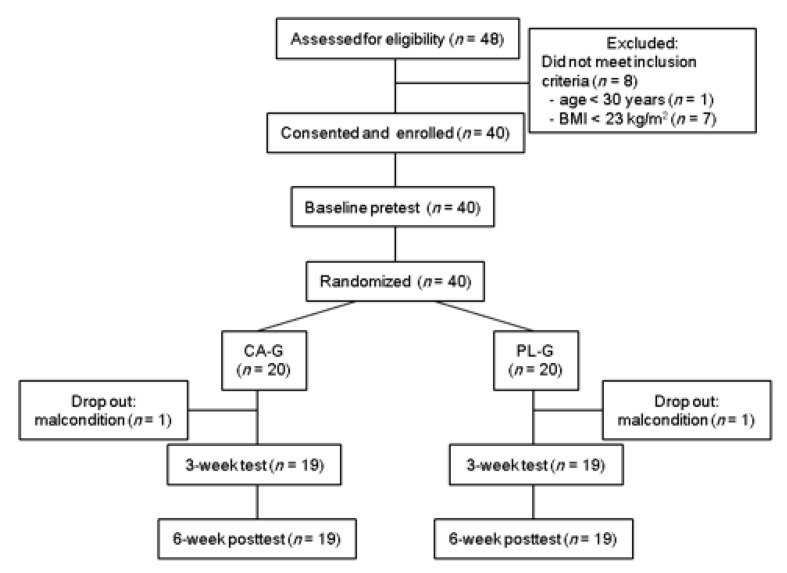
Flowchart for the selection of subjects. CA-G, capsinoid group; PL-G, placebo group.

We screened 48 volunteers for eligibility, and eight were excluded: one was aged <30 years and seven had BMI < 23 kg/m^2^ (Figure 1). Therefore, 40 subjects were enrolled in this study, pretested, and randomly allocated to CA-G (*n* = 20) or PL-G (*n* = 20). Two subjects dropped out of this study. Therefore, data were analyzed for 38 subjects. Anthropometric, [total-Hb]_sup_, and metabolic parameters and the average room temperature at the start and the end of measurements for each measurement taken for all participants are shown in Table 1. No significant differences between CA-G and PL-G at each measurement point were observed. There were no significant intervention interactions (group × time, *p* = 0.468) and main effect on the group (*p* = 0.318), but did main effect on the time (*p* < 0.001) for the average room temperature. Unexpectedly, there were significant increases in the average room temperature between baseline and 3 or 6 weeks (*p* < 0.05 for 3 weeks and *p* < 0.001 for 6 weeks, respectively) in both the CA-G and the PL-G.

**Figure 2:**
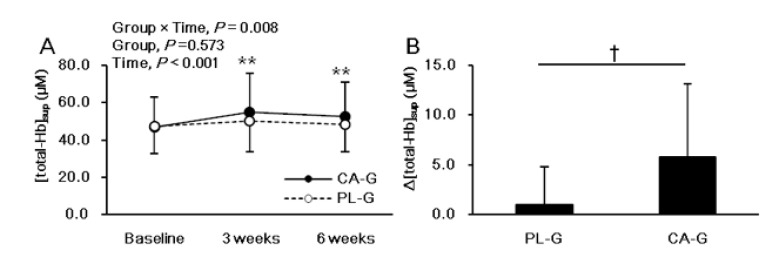
Mean (± SD) [total-Hb]_sup_, which is an indicator of brown adipose tissue vascular density in the supraclavicular region, at baseline and at 3 and 6 weeks after ingestion of capsinoid (●) and placebo (○) in all participants (*n* = 38) (**A**). Mean (± SD) changes in [total-Hb]_sup_ after the 6-week intervention in all participants (*n* = 38) (**B**). There were significant intervention interactions (group × time, *p* = 0.008) and main effect on the time (*p* < 0.001), but no main effect on the group (*p* = 0.573) by using ANOVA. The post hoc comparison between baseline and 3 or 6 weeks showed a significant increase in the [total-Hb]_sup_ (*p* < 0.001 for 3 weeks and *p* < 0.001 for 6 weeks, respectively) only in the CA-G (A; ** *p* < 0.01). A Welch test was conducted and it indicated that there was significant difference between the CA-G and PL-G (B; ^†^ *p* < 0.05). CA-G, capsinoid group; PL-G, placebo group; [total-Hb]_sup_, total hemoglobin concentration in the supraclavicular region.

Figure 2 shows the analysis results of [total-Hb]_sup_, which is an index of BAT-d. There were significant intervention interactions (group × time, *p* = 0.008) and main effect on the time (*p* < 0.001), but no main effect on the group (*p* = 0.573) for [total-Hb]_sup_. The post hoc comparison between 0 and 3 or 6 weeks showed a significant increase in the [total-Hb]_sup_ (*p* < 0.001 for 3 weeks and *p* < 0.001 for 6 weeks, respectively) only in the CA-G (Figure 2A). The change in [total-Hb]_sup_ during the 6-week period was significantly greater by 12.4% in CA-G than in PL-G (5.8 ± 7.3 versus 1.0 ± 3.8 µM; *p* = 0.017) (Figure 2B).

**Figure 3:**
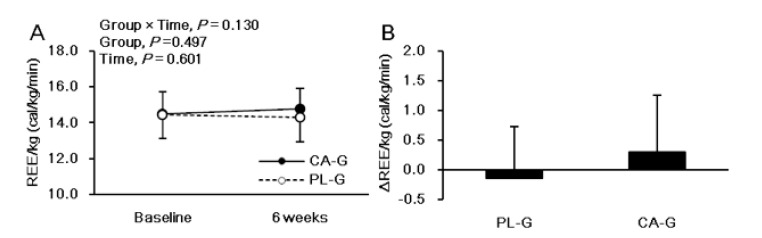
Mean (± SD) REE/kg at baseline and at 6 weeks after ingestion of capsinoid (●) and placebo (○) in all participants (*n* = 38) (**A**). Mean (± SD) changes in REE/kg after the 6-week intervention in all participants (*n* = 38) (**B**). There were no significant intervention interactions (group × time, *p* = 0.130) or main effects on the group (*p* = 0.497) or the time (*p* = 0.601) according to the ANOVA (**A**) results. A Welch test was conducted which indicated that there was a significant difference between the CA-G and PL-G (**B**). CA-G, capsinoid group; PL-G, placebo group; REE/kg, resting energy expenditure per kg.

**Figure 4:**
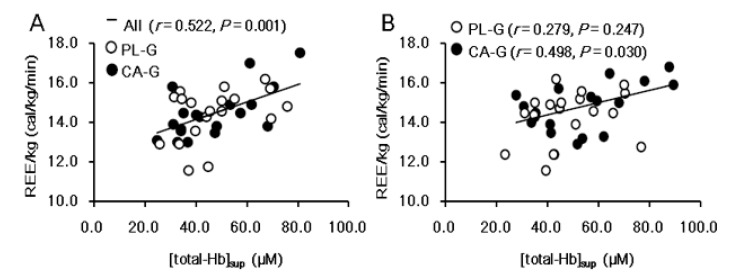
There were significant correlations between [total-Hb]_sup_ and REE/kg in all participants pre-supplementation (**A**) and only in the CA-G in post-supplementation (**B**). Pearson’s correlation coefficient is denoted by *r*. CA-G, capsinoid group; PL-G, placebo group; REE/kg, resting energy expenditure per kg; [total-Hb]_sup_, total hemoglobin concentration in the supraclavicular region.

Figure 3 shows the analysis of REE/kg. There were no significant intervention interactions (group × time, *p* = 0.130) or main effects on the group (*p* = 0.497) or the time (*p* = 0.601) for REE/kg. The REE/kg at 6 weeks after the intervention was higher in CA-G (14.8 ± 1.1 cal/kg/min = 1597.8 kcal/day) than in PL-G (14.3 ± 1.3 cal/kg/min = 1552.6 kcal/day), although the difference was not significant (*p* = 0.228) (Figure 3A). The change in REE/kg during the 6-week period was 0.3 ± 1.0 and −0.2 ± 0.9 cal/kg/min in the CA-G and PL-G, respectively (Figure 3B). The REE/kg changes in CA-G were 48.3 kcal/day higher than that of PL-G at the end of the 6-week intervention, a difference that was not significant (*p* = 0.130). We found a positive correlation between [total-Hb]_sup_ and REE/kg in all participants pre-supplementation (*r* = 0.522, *p* = 0.001) (Figure 4A) and in the CA-G post-supplementation (*r* = 0.498, *p* = 0.030) (Figure 4B).

**Figure 5:**
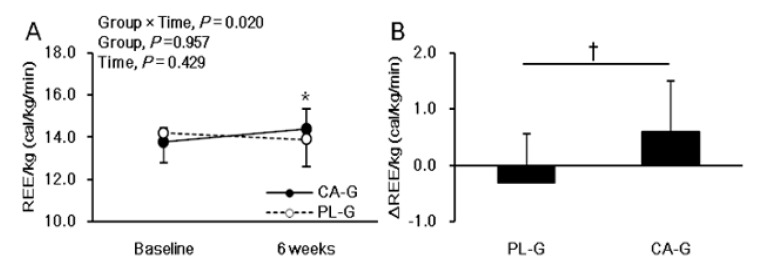
Mean (± SD) REE/kg at baseline and after 6-week ingestion of capsinoid (●) and placebo (○) in the overweight (BMI ≥ 25 kg/m^2^) subgroup (*n* = 24) (**A**). Mean (± SD) changes in REE/kg after the 6-week intervention in the overweight subgroup (*n* = 24) (**B**). There was a significant intervention interaction (group × time, *p* = 0.020); however, no main effects on the group (*p* = 0.957) or the time (*p* = 0.429) were observed by ANOVA. The post hoc comparison between baseline and 6 weeks showed a significant increase in REE/kg (*p* = 0.041) only in the CA-G (A; * *p* < 0.05). A Welch test was conducted which indicated that there was significant difference between the CA-G and PL-G (B; ^†^ *p* < 0.05). CA-G, capsinoid group; PL-G, placebo group; REE/kg, resting energy expenditure per kg.

For the subgroup with a BMI over 25 kg/m^2^, there was a significant intervention interaction (group × time, *p* = 0.020), but no main effect on the group (*p* = 0.957) or the time (*p* = 0.429) for REE/kg. The post hoc comparison between 0 and 6 weeks showed a significant increase in the REE/kg (*p* = 0.041) only in the CA-G among those with a BMI over 25 kg/m^2^. The REE/kg at the end of 6 weeks was greater in CA-G (*n* = 10, 14.4 ± 1.0 cal/kg/min = 1641.1 kcal/day on average) than in PL-G (*n* = 14, 13.9 ± 1.3 cal/kg/min = 1565.6 kcal/day on average), although the difference was not significant (*p* = 0.333) (Figure 5A). The changes in REE/kg at the end of 6 weeks were significantly greater (∆0.9 cal/kg/min = 108.7 kcal/day on average) in CA-G (0.6 ± 0.9 cal/kg/min = 80.4 kcal/day on average) than in PL-G (−0.3 ± 0.9 cal/kg/min = −28.2 kcal/day on average) (*p* = 0.021) in the subgroup with BMI over 25 kg/m^2^ (Figure 5B). In addition, VATA decreased more in CA-G (−12.2 ± 12.5 cm^2^) than in PL-G (0.5 ± 18.1 cm^2^), in the overweight subgroup, although the difference was not significant (*p* = 0.056).

In this study, two subjects dropped out during intervention because of mild and non-serious adverse events: one from CA-G who developed a subjective symptom of hemorrhoid, and the other from PL-G who had a malcondition after an overseas business trip. No other serious and/or severe adverse events were observed relating to capsinoid ingestion. Other safety measurements such as blood pressure and sympathetic nervous system did not change in subjects during the 6 weeks. In summary, there were no evident safety issues with continuous capsinoid ingestion.

## 4. Discussion

The main finding of this study is that BAT-d, as evaluated by [total-Hb]_sup_, significantly increased with daily ingestion of capsinoid during the first 3 weeks of intervention and remained elevated at the 6-week point in middle-aged, normal to overweight males. We also found a significantly positive correlation between BAT-d and REE/kg in all participants pre-supplementation and among those in the CA-G post-supplementation. Further, REE/kg in the overweight (BMI ≥ 25 kg/m^2^) subgroup significantly increased during the 6-week capsinoid supplementation. These results indicate that capsinoid supplementation could promote the increase in BAT-d for a healthy, middle-aged, and normal to overweight population in good correlation with REE.

Although not significant, there was a decrease in [total-Hb]_sup_ from the 3rd week to the 6th week not only in the CA-G, but also in the PL-G. However, a significant increase in BAT-d was observed but only in the CA-G at the 6th week. Taken together, although the effect of CA may not have been strengthened from the 3rd week to the 6th week, we confirmed that the effect was sustained at least to the end of the 6th week in this study and to the end of the 8th week in a previous study [27]. The average room temperature at the start and the end of measurements slightly, but significantly, increased from baseline to the 3rd week and 6th week in both groups for an unknown reason, which coincided with the increase in [total-Hb]_sup_ in the CA-G, which was physiologically an inverse direction. However, as we confirmed that [total-Hb]_sup_ was not different between temperatures of 19 °C versus 27 °C [2], a slightly increased room temperature from baseline to the 3rd week and 6th week in both groups would not influence [total-Hb]_sup_.

BAT activity declines with advancing age [6,7,8] and increasing body adiposity [7,8], and the responsiveness of BAT to cold or thermogenic ingredients has been extensively studied in younger generations [11,21]. Thus, knowledge regarding BAT activity and responsiveness according to TRP–SNS–BAT axis function in overweight and older individuals is still limited. In fact, despite unknown BAT characteristics, Galgani et al. observed an increase in REE (53 kcal/day on average) after combining the two dihydrocapsiate groups (3 and 9 mg/day) in a 4-week supplementation study of middle-aged individuals who were overweight and obese [22]. In addition, capsiate ingestion (10 mg/day) significantly increased REE (44 kcal/day on average) in middle-aged individuals who were overweight and obese [16]. In this study, REE/kg in the overweight (BMI over 25 kg/m^2^) subgroup significantly increased during the 6-week capsinoid supplementation (80 kcal/day on average). The amplitude of the increase in this study is comparable to, or marginally greater than, that reported in previous studies on dihydrocapsiate [22] and capsiate [16] supplementation. Therefore, the results of the current study are coherent with those of previous findings [16,22] and additionally provide new evidence supporting an increase in REE/kg for overweight subgroups. If participants in the overweight subgroup keep expending an extra 80 kcal/day through ingestion of capsinoids over a year, based on the present study findings, they could consequently reduce 4.2 kg of body fat, assuming that all the EE comes from fat oxidation. Moreover, they may prevent progression to obesity and related diseases. However, they would still need to lead a healthy lifestyle, otherwise they could easily ruin an extra 80-kcal/day EE if they consume high-calorie foods.

We found that there was a positive correlation between BAT-d and REE/kg pre-supplementation in all participants and post-supplementation only among those in the CA-G group. The reason for the lack of a correlation between BAT-d and REE/kg post-supplementation in the PL-G is unknown. This is a reasonable observation, considering that BAT is involved in adaptive thermogenesis [11]. However, REE/kg did not exhibit a significant difference between groups in contrast to BAT-d that did. This is probably because the amplitude of the BAT-d increment after the supplementation was relatively small (12%) in the middle-aged, normal to overweight participants. A previous study conducted among young lean individuals reported BAT-d increase by 47% [27], although no REE/kg was measured. Another possible reason for not finding a significant difference in REE/kg, despite the elevation in BAT-d, might have been the time delay from BAT activation to increased tissue mitochondrial respiration. A more prolonged supplementation may yield a significant increase in both BAT-d and REE/kg.

The post-supplementation increase in REE/kg found in the overweight subgroup might have been partly due to additional pathways to the TRP–SNS–BAT axis, such as muscle and WAT activation. Previous studies indicate that capsinoids increase uncoupling protein 1 (UCP1) mRNA levels in BAT, UCP2 mRNA levels in epididymal fat, and UCP3 mRNA levels in skeletal muscle [44,45,46]. Hence, it is speculated that the browning of subcutaneous fat and/or increase in skeletal muscle metabolism is related to the elevated REE/kg found in the overweight subgroup in this study. However, we failed to detect any significant increase in VO_2_, either in the forearm muscle or in the subcutaneous adipose tissue. The reason for the lack of changes in these tissues may be the location of the measurement, which should be considered in future studies.

Previous animal studies have revealed that selective activation of TRPV1 channels located in the upper gastrointestinal tract triggers the thermogenic reflex through the intermediary step of activating vagal afferents and sympathetic efferents innervating the BAT through the central thermogenic neurons. The efferent SNS signal is transmitted to the nerve ends, from which noradrenaline is released. Subsequently, noradrenaline binds to β-adrenergic receptors on the brown adipose cells, thereby activating UCP1 on the inner mitochondrial membrane and resulting in lipid mobilization and the induction of BAT thermogenesis [15,47,48]. In this context, we monitored resting LF/HF, which is a reflection of SNS activity to test our hypothesis that resting LF/HF would be higher in individuals with higher BAT-d. A previous study reported that body weight loss due to the repeated intake of capsinoids (CH-19 Sweet) was significantly correlated to the SNS response after ingesting capsinoids with food [21]. However, we failed to identify any significant differences in resting LF/HF between the two groups, presumably because of a different study design from that used in the previous study, that is, lack of diet-induced thermogenesis in this study. We measured VO_2_ in the muscle and adipose tissue; however, we found no significant difference in these parameters between the two groups. The reason for the marginal difference in elevated whole-body VO_2_ induced by capsinoid intake between the supplement and placebo groups is possibly that small amounts of local muscle and fat tissue were analyzed.

We selected the supraclavicular fossa as the measurement location because this region showed one of the highest BAT in the FDG-PET/CT studies [5,49,50,51]. Despite the fact that the supraclavicular fossa contains different types of tissues (the subclavian vessels, brachial plexus, omohyoid and scalene muscles, fat, lymph nodes, and posterior lung apex), we have accumulated evidence to show that [total-Hb]_sup_ could be a measure of BAT-d. Although [total-Hb]_sup_ reflects microvascular bed of sum of abovementioned different tissues, we believe that the basis for the application of NIR_TRS_ to assess BAT properties is that the microvascular bed—as evaluated by [total-Hb]_sup_—is more abundant in BAT than in WAT [31]. The [total-Hb]_sup_ measures were compared under thermoneutral and cold conditions. As there was no difference in the [total-Hb]_sup_ between the two conditions [2], indicators determined by NIR_TRS_ were used without cold exposure in this study. Significant correlations were found between [total-Hb]_sup_ and^18^FDG–PET/CT indicators [2] and between [total-Hb]_sup_ and thermogenesis [3]. Studies on capsinoids [27] or catechin [24] supplementation revealed a significant increase in [total-Hb]_sup_. Upon withdrawal of capsinoids supplement, a decrease in [total-Hb]_sup_ was observed [27]. Thus, we believe that NIR_TRS_ is a new approach for evaluating BAT-d. Nonetheless, studies on the validity of [total-Hb]_sup_ are limited; therefore, this validity should be examined by future investigations [4]. Note that this NIR_TRS_ methodology does not share principles with infrared thermography [52] or conventional thermography [53], which possess several limitations. Specifically, heat emission from the intrascapular area was not due to BAT thermogenesis, but to blood flow changes and the low insulation capacity of the thin subcutaneous fat layer.

There are several limitations in this study. First, bioelectric impedance is far from being the most accurate way to assess body composition as there is great variability with this technique and since it is affected by several situations. Intervention studies require greater accuracy so further research using densitometry will be needed to assess body composition. Second, although we instructed participants not to change dietary patterns during the intervention, we did not measure the dietary patterns during the intervention.

## 5. Conclusions

In conclusion, capsinoid supplementation promoted increases in [total-Hb]_sup_, a reflection of BAT-d, showing a good correlation with REE for a healthy, middle-aged, and normal to overweight population. The results of this study indicate that prolonged capsinoid intake would be helpful for maintaining and improving metabolic health status in middle-aged, normal to overweight populations through BAT enhancement.

## Figures and Tables

**Table 1:** Subject profiles and the average room temperature at each measurement point.

	Capsinoid (*n* = 19)			Placebo (*n* = 19)		
	Baseline	3 Weeks	6 Weeks	Baseline	3 Weeks	6 Weeks
Age (years)	43.5 ± 7.9	-	-	43.1 ± 8.0	-	-
Body weight (kg)	75.1 ± 7.4	75.7 ± 7.4	75.1 ± 7.4	75.5 ± 7.4	76.0 ± 7.4	75.9 ± 7.4
BMI (kg/m^2^)	25.2 ± 1.4	25.3 ± 1.5	25.2 ± 1.6	25.7 ± 1.3	25.8 ± 1.3	25.8 ± 1.3
VATA (cm^2^)	76.1 ± 34.9	71.9 ± 27.9	71.0 ± 30.9	78.1 ± 32.8	78.0 ± 33.8	80.5 ± 31.5
[total-Hb]_sup_ (µM)	46.8 ± 16.0	55.1 ± 20.5 *	52.7 ± 18.6 *	47.2 ± 14.5	50.0 ± 16.3	48.2 ± 14.5
REE/kg (cal/kg/min)	14.5 ± 1.3	-	14.8 ± 1.1	14.4 ± 1.3	-	14.3 ± 1.3
SFOC (µM O_2_/min)	1.7 ± 0.8	1.0 ± 0.9	1.8 ± 1.5	1.5 ± 0.7	1.3 ± 0.8	1.3 ± 1.2
MOC (µM O_2_/min)	13.0 ± 4.7	13.4 ± 4.3	13.0 ± 5.4	13.4 ± 5.7	13.0 ± 4.6	12.6 ± 5.2
Average room temperature at the start and the end of measurements (°C)	23.4 ± 0.4	23.9 ± 0.7 *	24.4 ± 0.4 *	23.3 ± 0.5	24.2 ± 0.6 *	24.4 ± 0.5 *

MOC, muscle oxygen consumption; REE/kg, resting energy expenditure per kg; SFOC, subcutaneous fat oxygen consumption; [total-Hb]_sup_, total hemoglobin concentration in the supraclavicular region; VATA, visceral adipose tissue area. Data are expressed as the mean ± SD. [total-Hb]_sup_ is an indicator of brown adipose tissue vascular density in the supraclavicular region. There was significant difference for time in each group (* *p* < 0.05, baseline versus 3 or 6 weeks), but not for groups analyzed by ANOVA.
